# Early outcome of off-pump versus on-pump coronary revascularization

**DOI:** 10.11604/pamj.2014.17.309.1723

**Published:** 2014-04-22

**Authors:** Saeed Davoodi, Abbasali Karimi, Seyed Hossein Ahmadi, Mehrab Marzban, Namvar Movahhedi, Kyomars Abbasi, Abbas Salehi Omran, Mahmood Shirzad, Mehrdad Sheikhvatan, Seyed Hesameddin Abbasi, Payvand Bina

**Affiliations:** 1 Tehran Heart Center, Tehran University of Medical Sciences, Tehran, Iran

**Keywords:** Coronary artery bypass grafting, Off-pump, Cardiopulmonary bypass, Outcome

## Abstract

**Introduction:**

The use of coronary artery bypass surgery (CABG) with cardiopulmonary bypass (CPB) or without CPB technique (off-pump) can be associated with different mortality and morbidity and their outcomes remain uncertain. The goal of this study was to evaluate the early outcome of on-pump versus off-pump CABG.

**Methods:**

We conducted a retrospective database review of 13866 patients (13560 patients undergoing on-pump CABG and 306 patients undergoing off-pump CABG) at Tehran Heart Center between January 2002 and January 2007. We compared preoperative, operative, and postoperative characteristics between them.

**Results:**

In-hospital mortality in the on-pump group was 0.8% compared to 0.7% in the off-pump group (P=0.999) and in-hospital morbidity was 11.7% and 6.5%, respectively (OR: 1.533, 95%CI: 0.902-2.605, P=0.114). Postoperative atrial fibrillation was more prevalent in on-pump versus off-pump surgery (6.0% vs 3.0%, P=0.028), however there were no statistical significant differences in other postoperative complications with regard to cardiac arrest (P=0.733), prolonged ventilation (P=0.363), brain stroke (P=0.999), renal failure (P=0.525), and postoperative bleeding (P=0.999). The mean length of stay in hospital (P=0.156) and in ICU (P=0.498) was also similar between the two groups.

**Conclusion:**

The results from an Iranian population-based study showed similar early mortality and morbidity of off-pump CABG in comparison to on-pump surgery.

## Introduction

Recent trials have shown that the two techniques of off-pump and on-pump coronary artery bypass surgery (CABG) may lead to the different mortality and morbidity especially in high risk patient populations [1]. The different rates in morbidity in these two techniques have been observed in postoperative cognitive impairment, incidence of renal failure, blood loss, prolonged mechanical ventilation, and length of stay in hospital and intensive care unit [2–5]. In most previous studies, it was confirmed that off-pump CABG surgery is a safe and viable alternative to conventional CABG as a treatment modality for surgical coronary revascularization [6–9]. However, the usage of more blood products in on-pump technique and needs to contend with heart motion during off-pump surgery has been considerable. In some other studies, although off-pump surgery reduced perioperative morbidity, its similar outcome compared to on-pump surgery is not clearly determined [9].

While the statistics showed the benefits of off-pump surgery in different age groups and with different co-morbidities, some studies proved no specific harm due to cardiopulmonary bypass [6–10]. Besides, in some others, on-pump surgery has been associated with significant pulmonary complications and functional changes such as increase in lung vascular permeability that can result in the development of ARDS, atelectasis, alterations of lung function, and arterial blood gas imbalance [11–14].

According to the different results in the outcome of off-pump in contrast to on-pump surgery, we tried to evaluate the early outcome of on-pump versus off-pump CABG among Iranian population.

## Methods

Demographic and clinical characteristics of 13866 patients undergoing isolated CABG (13560 patients undergoing on-pump CABG and 306 patients undergoing off-pump surgery) from 1 January 2002 to 1 January 2007 were collected and entered into a computerized database. All patients with the history of concomitant cardiac and non-cardiac operations were excluded.

In this study, CAD was considered significant if there was a 75% or greater stenosis in the cross-sectional diameter and 50% or greater stenosis in the luminal view [15]. The following variables were collected for statistical analysis including the preoperative variables: 1) general characteristics: age, gender, and body mass index; 2) preoperative risk factors: current smoking history (patient regularly smokes a tobacco product/products one or more times per day or has smoked in the 30 days prior to admission) [16], hypercholesterolemia (total cholesterol ≥ 5.0 mmol/l, HDL-cholesterol ≥1.0 mmol/l in men, or ≥1.1 mmol/l in women, triglyceride ≥ 2.0 mmol/l) [17], family history of CAD (first-degree relatives before the age of 55 in men and 65 years in women) [18], hypertension (systolic blood pressure ≥140 mmHg and/or diastolic ≥90 mmHg and/or on anti-hypertensive treatment) [19], diabetes mellitus (symptoms of diabetes plus plasma glucose concentration ≥11.1 mmol/l or fasting plasma glucose ≥7.0 mmol/l or 2-hp ≥11.1 mmol/l) [20], renal failure (creatinine >355 µmol/l with a rise of >44 units or urine output below 0.3 ml/kg for 24 h), cerebrovascular disease, peripheral vascular disease, and chronic lung disease; 3) preoperative cardiac status: recent myocardial infarction (an acute event with abnormal creatine phosphokinase and troponin levels), Canadian Cardiovascular Society (CCS) score, arrhythmia, and previous CABG and PCI; and 4) preoperative homodynamic status: number of defected coronary vessels, left main disease ≥50%, and LVEF. The operative data included type of surgery (elective or emergency), the number of distal anastomoses with vein grafts, the use of internal mammary artery (IMA) as grafts, and the use of IABP.

We considered four criteria to a complicated postoperative short-term outcome: 1) in-hospital postoperative complications including existence of at least one of these complications: cardiac complications (heart block, cardiac arrest, postoperative bleeding and tamponade, and atrial fibrillation) and non-cardiac complications (brain stroke, transient ischemic attack, renal failure, urinary tract infection, pulmonary emboli, pneumonia, acute limb ischemia, multi-system failure, continuous coma ≥ 24 hours, and prolonged ventilation ≥10 hours); 2) prolonged stay in ICU before and after surgery; 3) prolonged length of stay in hospital (LOS) before and after operation; and 4) 30-day mortality rate (sometimes termed operative mortality) defined as death within 30 days of operation [21].

Results were reported as mean ± standard deviation (SD) for quantitative variables and percentages for categorical variables. The groups were compared using the Student′s t-test for continuous variables and the chi-square test or Fisher's exact test if required for categorical variables. The Analysis of Covariance (ANCOVA) was used as the multivariate analysis for the evaluation of differences in LOS between the two groups in presence of possible confounding factors. Multivariate logistic regression models for comparing postoperative atrial fibrillation and morbidity across the two groups in presence of confounders were established. Odds ratios (OR) and 95% confidence intervals (CI) for OR were calculated. P values of 0.05 or less were considered statistically significant. All the statistical analyses were performed using SPSS version 13 (SPSS Inc., Chicago, IL, USA) and SAS version 9.1 for Windows (SAS Institute Inc., Cary, NC, USA).

## Results

Demographic characteristics and preoperative clinical data are illustrated in Table 1. Among CAD risk factors, history of diabetes mellitus (P<0.001) and hypertension (P=0.004) was found more in on-pump group and the history of other risk factors were similar in the two groups. Previous PCI was more prevalent in the patients who underwent off-pump surgery (P=0.008). The mean preoperative ejection fraction was lower in on-pump group (P=0.002) and coronary artery involvement in these patients was more severe than off-pump group (P<0.001).

**Table 1:** Preoperative characteristics of patients undergoing on-pump and off-pump CABG

Characteristics	On-pump group (n=13560)	Off-pump group (n=306)	P value
Male gender	74.7	73.9	0.740
Body mass index (Kg/m^2^)	27.21±4.10	26.95±3.96	0.273
Age (year)	58.64±9.56	56.27±10.37	<0.001
Family history of CAD	36.7	33.2	0.214
Current cigarette smoking	39.1	38.9	0.968
Hyperlipidemia	67.4	64.6	0.309
Diabetes mellitus	31.9	21.3	<0.001
Hypertension	52.9	44.6	0.004
Renal failure	1.7	1.3	0.576
Last creatinine (mg/dl)	1.19±0.43	1.14±0.44	0.094
Recent myocardial infarction	39.1	38.6	0.862
Cerebrovascular disease	6.5	3.9	0.073
Peripheral vascular disease	1.8	1.6	0.817
Congestive heart failure	11.8	12.8	0.590
Arrhythmia	2.8	3.0	0.878
Previous CABG	0.2	0.0	0.999
Previous PCI	4.1	7.2	0.008
Ejection fraction (%)	49.40±10.28	51.34±10.19	0.002
CCS score	2.92±1.11	2.97±0.98	0.437
**Number of coronary arteries involvement**			<0.001
One vessel	4.2	60.3	
Two vessels	22.5	27.2	
Three vessels	73.3	12.5	
Left main lesions	9.5	3.0	<0.001

Data are presented as mean ± SD or percentages; CABG: Coronary Artery Bypass Grafting; PCI: Percutaneous Coronary Intervention; CCS: Canadian Cardiovascular Society Grading System

The urgency of the procedure in on-pump and off-pump groups were urgent in 14.1% and 9.7%, respectively (P=0.028). There were statistically significant differences between CABG performed with cardiopulmonary bypass or with beating heart with regard to Intra-mammary and radial arteries and also vein used as grafts (P < 0.001) (Table 2). Also, Intra-aortic balloon pump insertion was more frequent in on-pump group (P=0.020). Patients who underwent CABG with cardiopulmonary bypass had a mean pump time of 72.52±22.18 minutes and clamp time of 43.41±14.14 minutes.

**Table 2:** Operative characteristics of patients undergoing on-pump and off-pump CABG

Characteristics	On-pump group (n=13560)	Off-pump group (n=306)	P value
Emergency surgery	14.1	9.7	0.028
IABP insertion	2.4	0.3	0.020
IMA used as graft	98.8	95.1	<0.001
Number of IMA for graft	1.00±0.15	0.96±0.24	<0.001
Radial artery used as graft	10.6	2.9	<0.001
Anastomoses with venous grafts	98.4	61.8	<0.001
Number of vein for graft	2.54±0.90	0.79±0.78	<0.001
Blood transfusion	20.2	3.6	<0.001

Data are presented as mean ± SD or percentages; CABG: Coronary Artery Bypass Grafting IMA: Internal mammary artery; IABP: Intra-Aortic Balloon Pump

There were no significant differences between the two studied groups in postoperative complications (Table 3). Although, univariate analysis revealed more incidence of postoperative atrial fibrillation (P=0.028) and higher morbidity rate (P=0.005) in on-pump group, multivariate logistic regression analysis showed no significant differences in atrial fibrillation (OR: 1.736, 95%CI: 0.808-3.731, P=0.158) and morbidity rate (OR: 1.533, 95%CI: 0.902-2.605, P=0.114) between the two groups. Also, the mean LOS in hospital (P=0.156) and in ICU (P=0.498) were similar between the two groups in the analysis of covariance.

**Tableau 3:** Répartition des patients en fonction des aspects thérapeutiques et évolutifs

Paramètre	Effectif (n=130)	Pourcentage
Anesthésie		
Générale	62	47
Locale	68	52
**Techniques chirurgicales**		
Veau	45	34,6
Tennison	15	11,5
Millard	70	53,8
Résultats		
Excellent	70	53,8
Très bon	22	16,9
Bon	14	10,7
Satisfaisant	18	13,8
Mauvais	6	4,6
**Suites opératoires immédiates**		
Infections	2	1,53
Lâchage de fil	1	0,7
Simples	127	96,9
**Durée d'hospitalisation**		
1 jour	25	19,2
2 jours	14	10,7
3 jours	75	57,6
4 jours	9	6,9
5 jours	7	5,3

## Discussion

Some previous studies have reported improved in-hospital outcomes, similar completeness of revascularization, and shorter LOS in hospital with off-pump compared to conventional CABG [22]. In addition, off-pump surgery could reduce operative morbidity relative to on-pump CABG [23]. Some randomized controlled trials (RCTs) have been conducted, but these studies produced equivocal results and the quality and durability of these two techniques of revascularization remain poorly defined. We have evaluated and compared the short-term outcomes of patients who underwent CABG with these two techniques at our institution. In the present study, we found no significant differences in postoperative complications and in-hospital mortality between the two studied groups. Also, LOS in hospital and in ICU was similar between them. Previous studies in the comparison of short-term outcome of the two surgical techniques had considerably different results. Similar to our study, some recent studies failed to show significant benefit of CABG performed on the off-pump versus cardiopulmonary bypass [7, 10, 24, 25]. Some studies also showed slightly better outcome after off-pump CABG compared to on-pump surgery especially in some postoperative complications such as atrial fibrillation [1], renal dysfunction [26], and postoperative symptomatic transient psychotic syndromes [27] in the first techniques, whereas, most of the published studies from large databases showed an advantage of off-pump CABG over conventional method in terms of early morbidity and/or mortality [28–31]. It seems that the study and comparison of early outcome of the two techniques are not enough for selection of the best technique for patient who is candidate for isolated CABG and determination of long-term results of both techniques especially their impacts on patient's quality of life are necessary. Furthermore, other surgical indices such as operation time, surgeon's experience, and even preoperative and postoperative supportive programs should be considered.

In our study, although on-pump techniques was frequently used in high risk patients with more coronary vessels involvement, lower ejection fraction, and more CAD risk factors, but some other studies confirmed that off-pump CABG can be a better operative strategy in this subset of patients [32–34]. However, it has been also indicated that the proportion of the bypass grafts that were patent at three months was significantly lower in the off-pump group than in the on-pump group [35]. In the present study, we also showed that the use of arterial conduits were more in on-pump versus off-pump surgery. It may be resulted in better short- and long-term outcome of CABG that can be previously described [36–38].

## Conclusion

The present trial showed that off-pump CABG surgery had similar postoperative morbidity and mortality when compared to on-pump CABG. According to this similarity, it seems that on-pump procedure is still a gold standard surgery for patients who candidate for CABG. However, further studies with sufficient power are needed to evaluate the benefits of both studied surgical techniques in subgroups of high risk patients such as those with neurological, bleeding, or renal complications.
